# Home treatment in paediatric patients with Hunter syndrome: the first Italian experience

**DOI:** 10.1186/1824-7288-39-53

**Published:** 2013-09-09

**Authors:** Ferdinando Ceravolo, Italia Mascaro, Simona Sestito, Elisa Pascale, Antonino Lauricella, Elio Dizione, Daniela Concolino

**Affiliations:** 1 Department of Paediatrics, University “Magna Graecia” of Catanzaro c/o Ospedale Civile A.Pugliese, Viale Pio X, 88100 Catanzaro, Italy; 2 Present address: Operative Unit of Metabolic Disease, Bambino Gesù Children’s Hospital, Rome, Italy

**Keywords:** Hunter syndrome, Enzyme replacement therapy, Idursulfase, Mucopolysaccharidoses, Home treatment (max 6)

## Abstract

Hunter syndrome (mucopolysaccharidosis type II [MPS II], OMIM309900) is a rare X-linked lysosomal storage disorder caused by the deficiency of the enzyme iduronate-2-sulphatase, resulting in accumulation of glycosaminoglycans, progressive multisystem organ failure, and early death. Enzyme replacement therapy (ERT) with weekly intravenous infusions of idursulfase, a treatment for MPS II and commercially available since 2007, has been shown to improve certain symptoms and signs of the disease. The efficacy and safety data of this enzyme preparation have been widely reported and, after a change to the idursulfase *Summary of Product Characteristics* in March 2010, home ERT by infusion is now an option for selected patients. Previously reported experiences of home therapy in MPS II have shown increased treatment compliance and an improvement in quality of life for both patients and families. We report the results of the home therapy experience of 3 paediatric patients with MPS II in southern Italy. This pilot experience with home infusion is the first reported from Italy.

## Introduction

Hunter syndrome or mucopolysaccharidosis type II (MPS II) is an X-linked lysosomal storage disorder that is due to a deficiency of iduronate-2-sulfatase, an enzyme essential for the degradation and recycling of glycosaminoglycans (GAGs). This enzyme deficiency results in a progressive accumulation of GAGs in various tissues and organs. Clinical manifestations include severe airway obstruction, neurological decline, hepatosplenomegaly, skeletal deformities, and cardiomyopathy. Death usually occurs in the second decade of life [1].

Enzyme replacement therapy (ERT) for MPS II was approved first in the US in 2006 [2]. Six years of experience with this therapy has demonstrated the effectiveness of ERT in slowing the accumulation of GAGs in various organs such as the liver and spleen and in improving endurance as measured by by the distance walked in 6 minutes. However, no effects have been observed so far on the central nervous system. The treatment is invasive and onerous, involving weekly intravenous infusions over 4- to 5-hour periods, which results in serious inconvenience for patients and their families, who must spend a lot of time at the treatment centre and consequently face disruption to their normal life. In addition, in southern Italy the treatment centres where the therapy is administered are often far from where the patients live and therefore difficult or time consuming to reach. This has a negative impact on the family budget (travel expenses, workdays missed), on compliance with therapy, and on quality of life [3].

Given the positive reports of home therapy in patients with MPS II from other European and worldwide centres [4-7] and the ongoing successful experiences of home treatment in patients with Fabry disease in Italy, we expanded this care model to patients suffering from MPS II and followed them at our Hospital Unit. Here we report the results of our experience in southern Italy with 3 patients with Hunter syndrome who have been undergoing home treatment for 73 to 87 weeks.

## Patients and methods

Three patients affected with MPS II, aged between 6 and 14 years and treated in our centre with ERT, started home therapy with idursulfase (Table 1). Clinical phenotype of the patients was classified according to cognitive impairment. All patients showed stable disease, had experienced at least 12 months of previous ERT in hospital, and had not reported adverse drug reactions during the last 3 months of therapy. No patients needed pre-medication during home therapy. No patients had surgical support placement or severe respiratory failure (FEV <40%). Nursing staff were selected according to their technical skills and their competence in paediatric basic life support (PBLS) and treatment of adverse drug reactions. All nurses involved in the project received dedicated training in lysosomal storage disorders with a focus on MPS II. They were also trained in medication handling, storage, reconstitution, and administration, as well as in potential adverse event recognition and management. Each patient was assigned to a specific nurse who had experience with enzyme infusion treatment under physician supervision. Nurses met with the patients’ families and agreed on the date and time of the first home infusion. All nurses were equipped with a pump and infusion kits, monitors for assessment of vital signs, oxygen, and first aid drugs for adverse events.

**Table 1:** Infusion therapy in patients with MPS II

	**Patient 1**	**Patient 2**	**Patient 3**
Age at diagnosis	4 y 9 mo	1 y 8 mo	7 y 8 mo
Phenotype	severe	severe	mild
Age at start of ERT	4 y 11 mo	2 y 5 mo	11 y 3 mo
ERT at hospital (number of infusions)	69	157	128
ADRs at hospital	0	2	1
Age at start of ERT at home	6 y 4 mo	6 y 2 mo	16 y 2 mo
ERT at home (number of infusions)	87	73	74
ADRs at home	0	0	0

*ADR* adverse drug reaction, *ERT* enzyme replacement therapy.

In order to manage any home treatment–related complications that might arise, a “security care network” was set up for patients. The main components of this network were the reference centre, nurses, patients’ families, family doctors, and the patients’ local hospitals. The reference centre (University Pediatrics Department. Rare and Metabolic Diseases Unit. Magna Graecia University – Catanzaro) acted as coordinator and approver. The start date for infusions was decided on the basis of clinical data as reported by the nurse. Each patient’s family doctor was alerted in case of illness or when the nurses or the reference centre believed it to be appropriate. The local hospital was ready to intervene in case of severe drug adverse reactions. The reference centre received a clinical report each month from the nurses in order to follow the clinical evolution of the patients’ disease.

Compliance was the main parameter affecting effectiveness which was evaluated for home therapy. The frequency of infusions missed during home treatment was compared with the frequency of missed infusions observed for hospital treatment. Safety parameters were also recorded. These included number and type of adverse reactions, and number of missed infusions reported to the home health-care team. Parents were asked to complete a quality-of-life form using a validated questionnaire (EQ-5D) at the start of the treatment and after 1, 3, and 6 months. EQ-5D is a standardized instrument used as a measure of health outcomes [8,9].

The financial impact of home treatment was also examined, particularly with regard to associated travel costs and reductions in income due to missed workdays.

The project was approved by the ethics committee of the Ospedale Civile Pugliese Ciaccio.

## Results

After appropriate counselling concerning the potential risks and benefits, provided by the reference centre, the parents of all 3 patients agreed to home infusion.

Table 1 shows patient profiles and the number of infusions administered at home and at the hospital. During the first year of home treatment, more infusions were administered at home than had been administered at the specialised centre in the year previous to starting home treatment. Patients 1, 2, and 3 received 52, 52, and 51 infusions at home, respectively, compared with 49, 48, and 49 infusions at the hospital the year before. Also, the number of missed infusions was higher when the patients went for hospital treatment (3, 4, and 3 infusions were missed by patients 1, 2, and 3, respectively). This compares with only 1 missed infusion (due to temporary sickness in patient 3) when on home treatment. Reasons for missed infusions at the hospital included illness of patients or caregivers, and transportation problems.

The EQ-5D proxy scores of the patients’ parents increased when the patient was on home treatment, indicating an important improvement in quality of life. For families 1, 2, and 3 the baseline EQ-5D scores were −0.352, +0.011, and −0.181, respectively. After 1 month the scores were +0.137, +0.516, and +0.110, and at 6 months they were +0.367, +0.746, and +0.746 for families 1, 2, and 3, respectively.

Cost analysis revealed that home treatment resulted in an average savings of 11,000 euros per family during the study (range: 9,048–12,324 euros). Of this amount, 41% (range 28%–47%) was attributable to the costs of weekly travel from home to the hospital, and the remaining 59% (range 54%-72%) to family members not missing workdays.

No adverse reactions were reported during 234 home infusions.

## Discussion

Our experience demonstrates that home treatment with Elaprase is feasible and safe for selected patients with MPS II, as has been reported in other European and worldwide centres [4,6]. A significant benefit of this reported home therapy is that patients showed an almost perfect compliance with the treatment schedule. Home therapy is effective in helping to avoid missed infusions due to non-clinical reasons and helps to guarantee an adequate infusion schedule since it increases scheduling flexibility. Home therapy also helps to alleviate the burden of lifelong therapy in these patients and their families, as demonstrated by the improved quality of life shown by the increased EQ-5D proxy questionnaire scores. This occurred mainly in the areas of "usual activity" and "anxiety/depression" demonstrating that home care was perceived as less invasive and more compatible with the daily activities. According to this data, home treatment, appears to give families and patients a better perception of the disease. Obviously, given the functional and cognitive limitations of patients with Hunter disease it was unlikely to expect further improvements in the areas of "self care", "mobility" and "pain/discomfort".

Cost analysis shows that home therapy also lowers the impact of ERT on the family budget.

In conclusion, on the basis of our experience, home therapy should be a clear and logical choice for the management of selected MPS II patients, including those without severe chronic obstructive airway disease (FEV < 40%) who have been treated with ERT for at least 6 months at the hospital and have been without adverse drug reactions for the last 3 months.

## Competing interests

The authors declare that they have no competing interests.

## Authors’ contributions

FC analyzed the data and drafted the manuscript. IM, SS and EP have contributed in data analysis. AL and ED collected the data. DC analyzed the data and corrected the draft for final submission. All authors approved the final manuscript and share responsibility for what is published.
